# Polyphenol-rich Chinese olive extracts attenuate lipid accumulation in HepG2 cells, accompanied by AMPK phosphorylation and miRNA alterations

**DOI:** 10.3389/fnut.2026.1749622

**Published:** 2026-02-11

**Authors:** Hong Wang, Bingqi Huang, Wenhong Zhao, Gongliang Liu, Weidong Bai

**Affiliations:** 1 Guangdong Provincial Key Laboratory of Lingnan Specialty Food Science and Technology, College of Light Industry and Food Technology, Zhongkai University of Agriculture and Engineering, Guangzhou, China; 2 Academy of Contemporary Agricultural Engineering Innovations, Zhongkai University of Agriculture and Engineering, Guangzhou, China

**Keywords:** AMPK phosphorylation, Chinese olive, HepG2 cells, lipid accumulation, microRNA expression, polyphenol-rich extracts

## Abstract

**Introduction:**

Chinese olive (*Canarium album* L.) is a rich source of phenolic compounds and has been suggested to exhibit distinct lipid-lowering bioactivities from those reported for Mediterranean olive polyphenols.

**Methods:**

This study investigated the effects of polyphenol-rich Chinese olive extracts on sodium oleate-induced lipid accumulation in HepG2 cells.

**Results:**

Treatment with extracts was associated with a significant reduction in intracellular total lipid and triglyceride levels in a concentration-dependent manner. RT-qPCR results revealed that extract treatment was accompanied by the downregulation of lipogenesis-related genes (SREBP-1c, ACC1, FASN, and DGAT2) and the upregulation of *β*-oxidation-associated genes (PGC-1α, PPARα, CPT-1A, and ACOX1). Western blot analysis showed that extract treatment was associated with AMPK phosphorylation, occurring concurrently with the observed lipid-associated changes. Furthermore, treatment with extracts was accompanied by decreased expression of miR-122 and miR-21, which correlated with the expression of their respective lipid-metabolism target genes. UPLC–MS/MS analysis identified 39 phenolic compounds in the extract, including methyl brevifolincarboxylate and ellagic acid derivatives, indicating a complex phenolic composition.

**Discussion:**

These results demonstrate that attenuation of lipid accumulation by polyphenol-rich Chinese olive extracts in HepG2 cells is accompanied by AMPK phosphorylation and miRNA modulation. This finding supports the potential of Chinese olive extracts as a natural hepatoprotective ingredient for the development of functional foods and nutraceuticals.

## 1 Introduction

The liver serves as a central hub for complex biochemical processes, including the oxidation of triglycerides and the synthesis of lipoproteins and phospholipids, which are essential for metabolizing various nutrients (1). Dysregulation of lipid metabolism in the liver often leads to lipid accumulation, which is associated with several chronic diseases, including hyperlipidemia, non-alcoholic fatty liver disease (NAFLD), metabolic syndrome, and cardiovascular disease. To mitigate the risk of these diseases, it is crucial to address the dysregulation of lipid metabolism and reduce the deposition of excess fat (2).

Key enzymes and transcriptional regulators such as fatty acid synthase (FASN), acetyl-CoA carboxylase 1 (ACC1), and sterol regulatory element-binding proteins (SREBPs) play pivotal roles in lipogenesis. Their expression levels of these regulators fluctuate in response to changes in cellular energy status, highlighting their role in maintaining lipid homeostasis (3). Hepatic lipolysis is primarily regulated by several enzymes and transcription factors, such as adenosine monophosphate-activated protein kinase (AMPK), peroxisome proliferator-activated receptors *α* (*PPARα*), carnitine palmitoyltransferase 1A (*CPT-1A*) and diacylglycerol acyltransferase 2 (*DGAT2*), all of which integrate nutritional and hormonal signals to regulate lipid turnover.

MicroRNAs (miRNAs) have been identified as pivotal regulators of hepatic lipid metabolism, complementing the roles of enzymes and transcription factors (4). These small non-coding RNAs bind to target mRNAs to inhibit translation or promote degradation, thereby fine-tuning lipid metabolic pathways. Among them, miR-122, a liver-specific miRNA, exerts a significant influence on lipid homeostasis by directly suppressing genes involved in fatty acid oxidation and lipid synthesis, leading to lipid accumulation. AMPK and PPAR family members have been identified as downstream effectors of miR-122-mediated regulation. Transcriptomic analyses in mice revealed that transient sequestration of miR-122 downregulated genes involved in fatty acid metabolism and cholesterol biosynthesis, including the rate-limiting enzyme 3-hydroxy-3-methylglutaryl-CoA reductase. Similarly, inhibition of miR-21 expression enhances PPARα mRNA levels and improves mitochondrial dynamics, thereby reducing hepatic lipid deposition (5). The selection of miR-122 and miR-21 as investigative targets in the present study is based on their complementary functions as master regulators of lipid metabolism. Specifically, miR-122 promotes lipogenesis by targeting *SREBP-1c* and *DGAT2*, while miR-21 suppresses *PPARα*-mediated *β*-oxidation via conserved binding to its 3′UTR (6). Targeting both pathways simultaneously offers a comprehensive approach to rebalancing lipid synthesis and catabolism.

In recent years, numerous plant-derived polyphenolic compounds have emerged as potential inhibitors of lipid accumulation, demonstrating the ability to inhibit lipid synthesis while promoting lipid oxidation (7). Chinese olive [*Canarium album* (Lour.) Rauesch.] has a long history of cultivation and consumption in southern China, particularly in Fujian, Guangdong, Guangxi, and Yunnan provinces, as well as in Vietnam, Japan, and the Malay Peninsula. Unlike *Olea europaea* L., which is primarily cultivated for olive oil production, Chinese olive is consumed fresh and is rich in diverse bioactive components exhibiting anti-inflammatory, anti-diabetic, hepatoprotective, and gut microbiota-regulating properties (8). Previous research demonstrated that polyphenol-rich Chinese olive extract ameliorate hepatic lipid accumulation and is accompanied by AMPK activation *in vitro* and *in vivo* (9). Building on this work, our study uses an in vitro HepG2 steatosis model to provide additional descriptive evidence and to examine associated changes in selected miRNAs and lipid-metabolism transcripts.

Chinese olive extract consist of a broad spectrum of bioactive molecules—including phenolic acids, flavonoids, and phenylpropanoids—that are widely regarded as the principal contributors to olive-associated health benefits (8). Among these, phenolic acids, such as gallic acid, ellagic acid, and salicylic acid represent the predominant components (10). Flavonoids, another major subclass, include hyperoside, isoquercitrin, luteolin-7-*O*-glucoside, quercetin, and kaempferol, while additional constituents such as lignans, coumarins, and terpenoids further enrich the phytochemical complexity of Chinese olives (11). Chemically, polyphenol-rich Chinese olive extract differ markedly from Mediterranean olive (*Olea europaea*) polyphenols, with notably higher concentrations of methyl brevifolincarboxylate and ellagic acid derivatives (10). These compounds exhibit superior AMPK-activating potential compared with hydroxytyrosol-rich Mediterranean extracts, suggesting region-specific bioactive advantages (12). Nevertheless, few studies have systematically correlated the compositional characteristics of Chinese olive extract with their lipid-lowering mechanisms.

Accordingly, the present study aimed to investigate the lipid-lowering effects of Chinese olive extract, enriched in polyphenols (OP), in sodium oleate–treated HepG2 cells, focusing on changes in selected miRNAs and AMPK signaling. In addition, ultra-performance liquid chromatography coupled with tandem mass spectrometry (UPLC–MS/MS) was employed to characterize the phenolic profile of Chinese olive extract and to highlight candidate compounds potentially related to its lipid-regulatory activity.

## 2 Materials and methods

### 2.1 Materials and reagents

Ripe fruits of the Chinese olive variety ‘Tanxiang No.1’ were collected in September 2022 from Puning, Guangdong Province, China (116°09′58″E, 23°17′53”N). The fruits were thoroughly washed with water and pitted. Subsequently, the olives were crushed and stored at −20 °C for further analysis.

Dimethyl sulfoxide (DMSO) was purchased from Shanghai Aladdin Biochemical Technology Co., Ltd. (Shanghai, China). Fetal bovine serum (FBS), Dulbecco’s modified Eagle’s medium (DMEM) containing 4,500 mg/L glucose, penicillin G (100 units/mL) and streptomycin (100 μg/mL) were purchased from Thermo Fisher Scientific Inc. (Massachusetts, United States). TRNzol Universal Reagent, the miRNAcute miRNA Isolation Kit, FastKing gDNA Dispelling RT SuperMix, the miRNA 1st Strand cDNA Synthesis Kit, TIANScript II RT Kit and RNase-free ddH_2_O were purchased from TIANGEN Biotech Co., Ltd. (Beijing, China). SYBR Green Master Mix was procured from Nanjing Vazyme Biotech Co., Ltd. (Nanjing, China). Oil Red O and the BCA protein assay kits were purchased from Beyotime Biotech Inc. (Shanghai, China). Other reagents were of analytical grade. The Triglyceride (TG) assay kit was purchased from Nanjing Jiancheng Bioengineering Institute (Nanjing, Jiangsu, China). Rabbit anti-phosphorylated AMPK alpha (Thr172; pAMPK) and rabbit anti-AMPK primary antibodies were purchased from Affinity Biosciences (OH, United States). Rabbit anti-*β*-actin primary antibody was purchased from ABclonal Biotechnology Co., Ltd. (MA, United States). HRP-conjugated secondary antibody was purchased from Jackson ImmunoResearch Laboratories, Inc. (PA, United States). Other reagents were of analytical grade.

### 2.2 Preparation of polyphenol-rich Chinese olive extract

OP was prepared using an ethanol-based extraction procedure adapted from published approaches (13), with modifications optimized to enhance extraction efficiency and maximize phenolic yield. Briefly, olives were extracted with 70% ethanol at a ratio of 1:25 (w/v) using ultrasound (370 W for 20 min) followed by microwave treatment (500 W for 3 min). The extracts were centrifuged at 6000 rpm for 5 min and the supernatants were collected. Solvent was removed by rotary evaporation and the residue was freeze-dried to yield the extract used in this study. We note that extraction conditions can influence chemical composition; therefore, our conclusions are specific to this preparation.

### 2.3 Cell culture

HepG2 cells, obtained from the Cell Resource Center at the Institute of Basic Medical Sciences (Beijing, China), were cultured in DMEM supplemented with 10% (v/v) FBS and 1% (v/v) penicillin–streptomycin. The cells were incubated at 37 °C in a 5% CO_2_ incubator (Esco Technologies Pty Ltd., Singapore).

### 2.4 Cell cytotoxicity assay

The viability of HepG2 cells was assessed using the methylene blue assay (14). The cells were inoculated into 96-well plates at a concentration of 2.5 × 10^5^ cells per well and cultured at 37 °C with 5% CO_2_ for 24 h. The growth medium was then replaced with sodium oleate at concentrations of 100, 200, 300, 400, 500, 600, 700, 800, 900 and 1,000 μM, and the cells were cultured for an additional 24 h. Following this, 50 μL of methylene blue solution (composed of HBSS, 1.25% (v/v) glutaraldehyde and 0.6% (w/v) methylene blue) was added and incubated at 37 °C for 1 h. Subsequently, 100 μL of elution buffer (50% ethanol, 49% PBS and 1% acetic acid; v/v/v) was added to each well and shaken for 15 min. The cell viability for each treatment was expressed as a percentage of the absorbance value at 595 nm of the treated cells divided by that of the control group.

### 2.5 Cell treatments

Sodium oleate solution was prepared according to the method described by Zhang et al. (15) with minor modifications. The experimental treatments were as follows: (1) normal group (control); (2) SO-Treated group treated with 400 μM sodium oleate (SO-Treated); (3) low-concentration OP group treated with 400 μM sodium oleate and 50 μg/mL OP (OP50); (4) medium-concentration OP group treated with 400 μM sodium oleate and 100 μg/mL OP (OP100); (5) high-concentration OP group treated with 400 μM sodium oleate and 250 μg/mL OP (OP250). All groups were co-treated for 24 h at 37 °C in a 5% CO_2_ humidified atmosphere, after which the cells were collected for further study.

### 2.6 Oil red O staining and intracellular TG levels

HepG2 cells were washed three times with PBS and fixed with 4% formaldehyde for 30 min. Following fixation, the cells were washed with 60% isopropanol and stained with Oil Red O solution for 30 min at room temperature. The cells were then washed once with 60% isopropanol and subsequently with PBS. Fat droplets within the cells were observed using an inverted microscope (Nikon Eclipse, Melville, NY, United States). For quantification, 1 mL of isopropanol was added to each well to elute the stain, and absorbance was measured at 510 nm. Intracellular TG content was assessed using a commercial assay kit (Nanjing Jiancheng Technology Co., Ltd., Nanjing, China). Cell lysates were mixed with the enzyme working solution, incubated at 37 °C for 10 min, and absorbance was measured at 550 nm. A standard curve was prepared by serial dilution of the provided calibrator. TG concentration was calculated from the curve based on sample absorbance.

### 2.7 Western blot analysis

Total proteins were extracted from lysed cells using RIPA lysis buffer, and the protein content of the lysate was quantified using the BCA Kit. The proteins were separated by sodium dodecyl sulfate-polyacrylamide gel electrophoresis (SDS-PAGE) and subsequently transferred to a polyvinylidene difluoride (PVDF) membrane. The PVDF membrane was immersed in a PBS containing 5% skimmed milk powder and incubated on a shaker for 30 min. Primary antibodies were added and incubated overnight at 4 °C, including anti-AMPK (Rabbit mAb, 1:1,000), anti-pAMPK (Thr172; Rabbit mAb, 1:1000), and anti-*β*-actin (Rabbit mAb, 1:50,000). After three washes with PBS containing Tween 20 (PBST), the membranes were incubated with HRP-conjugated secondary antibodies (1:5,000) for 1 h at room temperature. Finally, enhanced chemiluminescence (ECL) detection reagent was employed to visualize the proteins. Western blot images were analyzed using Image-Pro Plus software.

### 2.8 Isolation of RNA from cultured cells and real-time qPCR analysis

Total RNA was isolated from HepG2 cells using the TRNzol Universal Reagent. The reverse transcriptase (RT) reaction (20 μM) was performed with 2 μg of total RNA, 4 μL of 5 × FastKing-RT SuperMix and RNase-free ddH_2_O. MicroRNA was extracted from HepG2 cells using the miRNAcute miRNA Isolation Kit, and the RT reaction (20 μM) was conducted using the miRNA 1st Strand cDNA Synthesis Kit, following the manufacturer’s instructions and employing a stem-loop method. Quantitative polymerase chain reaction (qPCR) detection was executed using SYBR Green Master Mix. The primers used for real-time PCR analysis were listed in Supplementary Tables S1, S2. The expression levels of genes and miRNAs were normalized to GAPDH and U6, respectively, and the expression of the respective genes or miRNAs was quantified using the 2^−ΔΔCt^ method.

### 2.9 Determination of phenolic composition by ultra-high performance liquid chromatography-tandem mass spectrometry

The phenolic composition was determined using an ExionLC™ UHPLC system coupled to a QTRAP^®^ 6,500^+^ triple quadrupole mass spectrometer (SCIEX, MA, United States). A Waters Acquity UPLC HSS T3 column (2.1 mm × 100 mm, 1.8 μm) was selected due to its moderate pH range (2.0–8.0) and ability to strongly retain highly polar organic compounds and metabolites. The mobile phase A was an aqueous solution containing 0.1% formic acid, and mobile phase B was 100% acetonitrile. The elution followed a linear gradient from 2 to 50% B for 0–10 min, 50 to 95% B for 10–11 min, remained at 95% B for 2 min (11–13 min), 95 to 2% B for 13–13.1 min, and finally remained at 2% B for 2 min. The flow rate was set at 400 μL/min, and the sample injection volume was 2 μL. The temperature of the column chamber was maintained at 40 °C. The ESI source conditions were set as follows: the capillary voltage was set at +5,500 V or −4,500 V in positive or negative mode, respectively; the curtain gas pressure was set at 35 psi; the temperature was set at 400 °C; the ion source gas pressure was set at 60 psi; and the DP was set at ±100 V.

### 2.10 Statistical analysis

All data were expressed as the mean ± standard deviation (SD, n = 3). Statistical analyses were performed using one-way analysis of variance (ANOVA), followed by Tukey’s multiple comparisons tests with IBM SPSS Statistics version 26 software. Differences were considered significant at *p* < 0.05.

## 3 Results and discussion

### 3.1 Effects of OP and sodium oleate at different concentrations on HepG2 cell viability

Given that HepG2 cells retain most hepatic metabolic functions (16), this study employed a sodium oleate-induced steatosis model to evaluate the lipid-lowering effects of OP *in vitro*. Prior to model establishment, the cytotoxicity of both OP and sodium oleate was assessed using the methylene blue assay.

**Figure 1:**
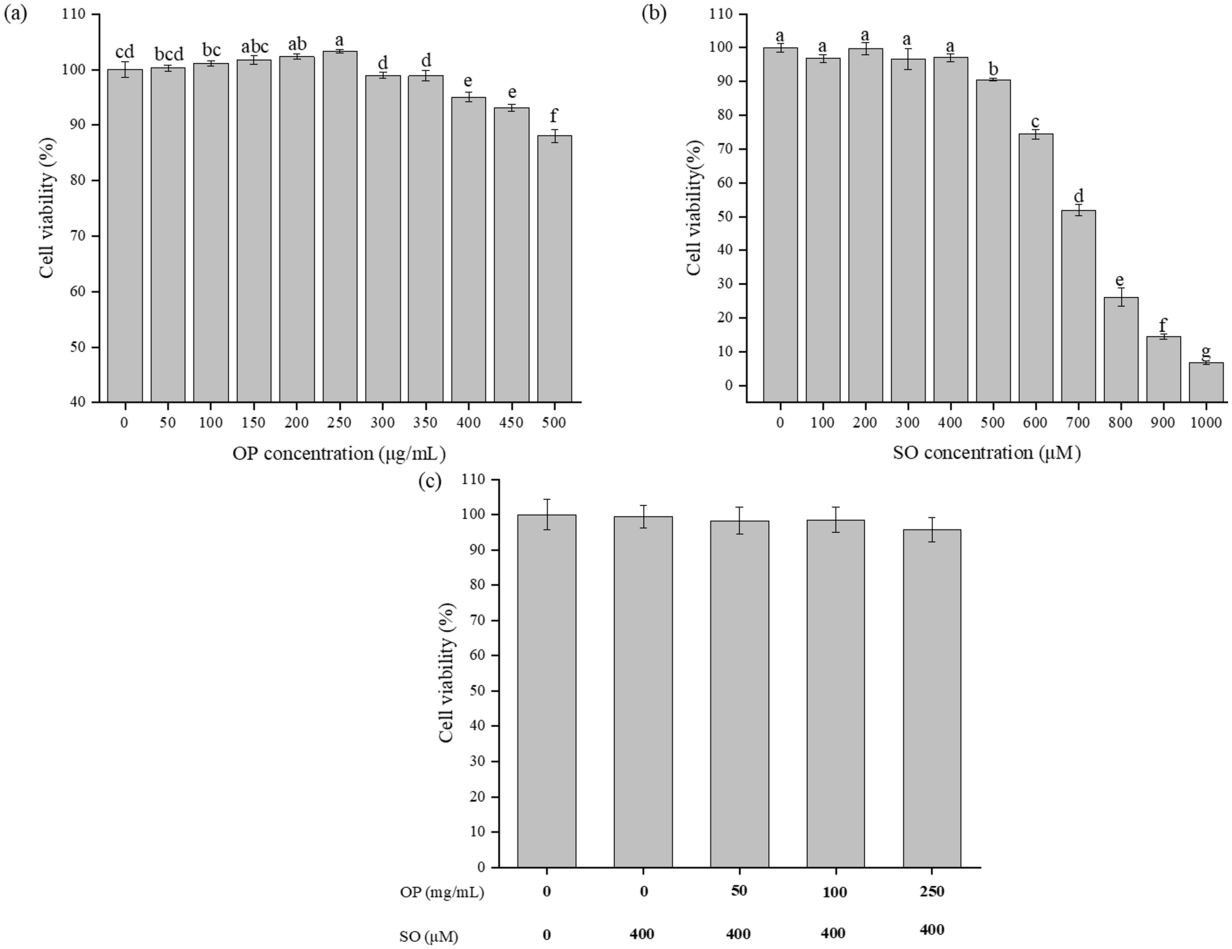
Effect of Chinese olive extracts (OP, **a**), sodium oleate (SO, **b**), and their combined treatment **(c)** on HepG2 cell viability. Data in the bar graphs are expressed as mean ± SD and represent three biological replicates. Different letters in the graph indicate significant differences (*p* < 0.05).

As shown in Figure 1a, OP concentrations ranging from 50 to 250 μg/mL did not induce any significant decline in HepG2 cell viability (*p* > 0.05), indicating negligible cytotoxicity and confirming that these concentrations would not interfere with subsequent molecular experiments. However, concentrations exceeding 250 μg/mL led to a gradual decrease in cell viability. Figure 1b illustrated the cytotoxicity of sodium oleate, showing that cell viability remained comparable to that of untreated controls between 100 and 400 μM. Therefore, 400 μM sodium oleate was selected as the optimal concentration for model induction. Furthermore, to rule out cytotoxicity from the combined treatment, cell viability was assessed under co-treatment conditions (sodium oleate + OP). As shown in Figure 1c, no significant reduction in viability was observed across the tested concentrations. It should be noted that the OP concentrations used in this study (50–250 μg/mL) were optimized for an *in vitro* mechanistic investigation. While these concentrations may be higher than the typical physiological plasma levels that can be achieved through dietary intake alone, they were selected to produce measurable biochemical responses and to establish clear concentration -response relationships without inducing cytotoxicity.

### 3.2 OP attenuated lipid accumulation in sodium oleate-treated HepG2 cells

**Figure 2:**
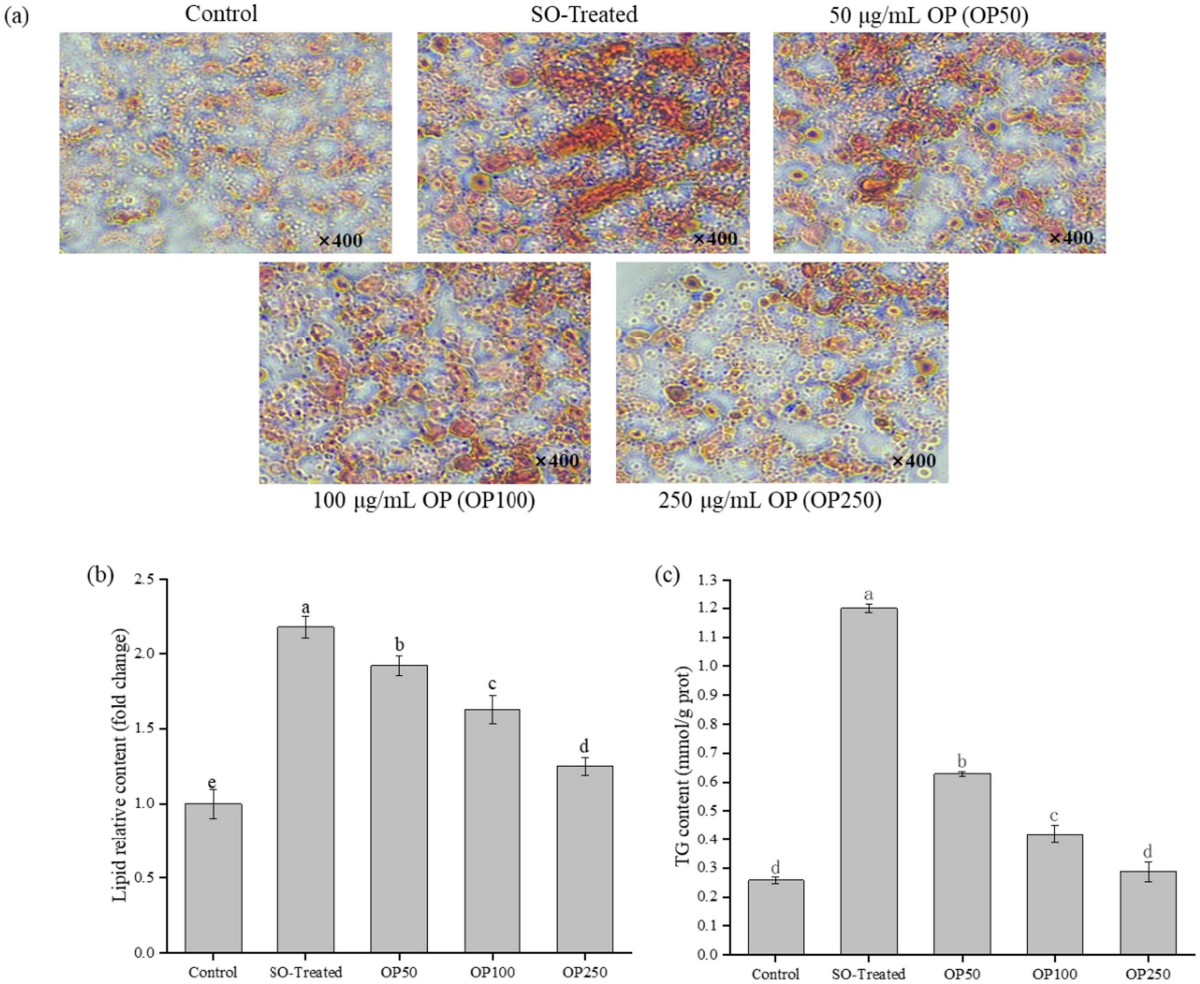
Effect of OP on lipid accumulation in SO-induced HepG2 cells. **(a)** Representative Oil Red O staining images of HepG2 cells (400 ×; SO-treated group: 400 μM sodium oleate-treated group); **(b)** Quantification of Oil Red O staining by colorimetric method; **(c)** Intracellular TG content. Data in the bar graphs are expressed as mean ± SD and represent of three biological replicates. Different letters in the graph indicate significant differences (*p* < 0.05).

To evaluate the effects of OP on intracellular lipid deposition, Oil Red O staining and triglyceride (TG) assays were performed. As shown in Figure 2a, minimal lipid droplets were observed in the control group, whereas the SO-Treated group exhibited a pronounced increase in red-stained cytoplasmic lipid droplets. Quantitative TG analysis (Figure 2b) revealed a fivefold increase in intracellular TG levels after 24 h of 400 μM sodium oleate induction compared to the control. However, OP treatment markedly reduced TG accumulation in a concentration-dependent manner.

Consistent results were observed in lipid staining (Figure 2c), where OP supplementation alleviated the sodium oleate–induced increase in intracellular lipid content (*p* < 0.05). These findings align with previously reported Oil Red O staining patterns in steatotic hepatocytes (9), collectively demonstrating the lipid-inhibitory potential of OP.

### 3.3 OP treatment downregulated lipogenic gene expression in sodium oleate-treated HepG2 cells

**Figure 3:**
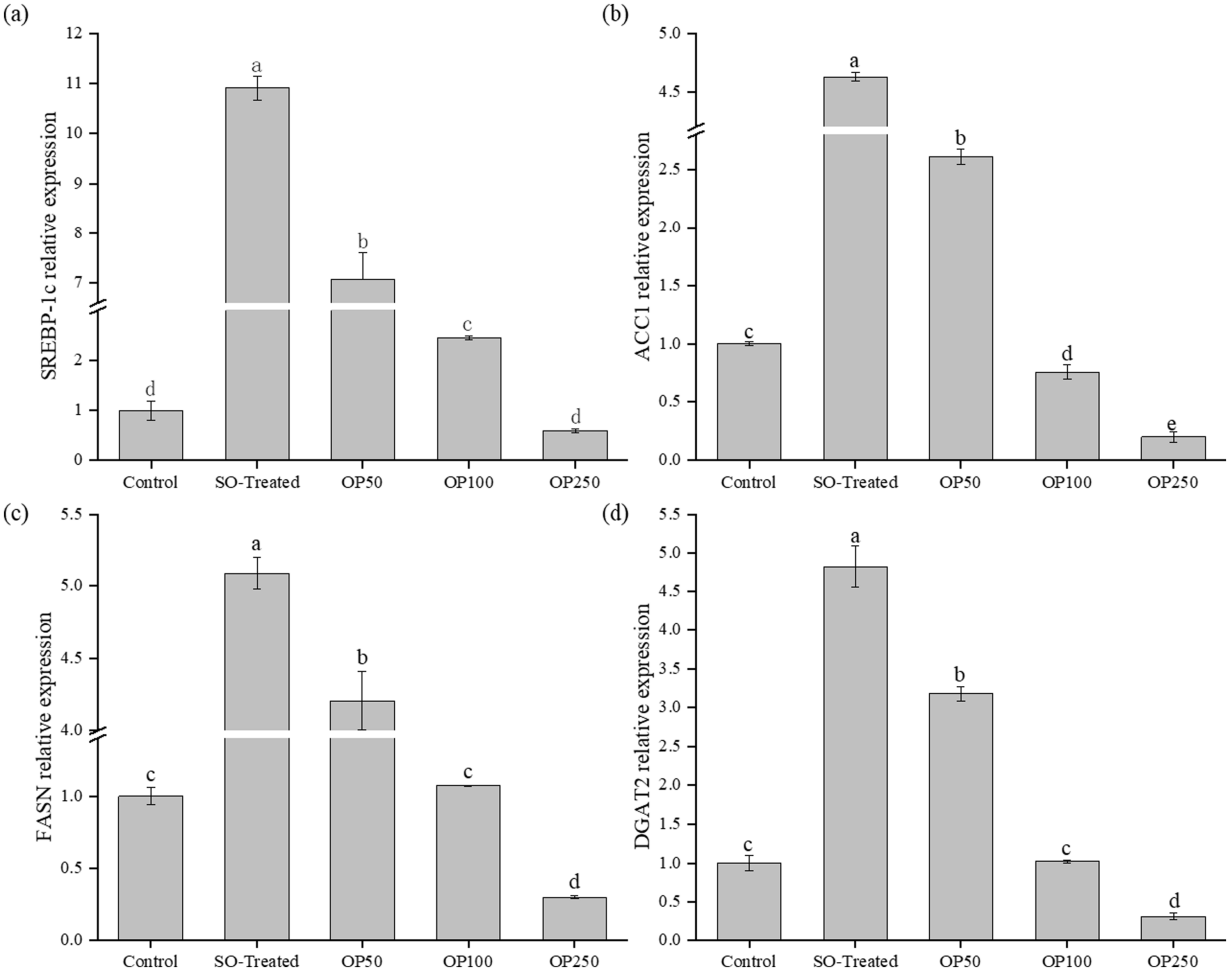
Effect of OP treatment on the relative expression of genes associated with fatty acid and triglyceride synthesis in SO-induced HepG2 cells. Relative transcript levels of SREBP-1c **(a)**, ACC1 **(b)**, FASN **(c)**, and DGAT2 **(d)**. Data in the bar graphs are expressed as mean ± SD and represent of three biological replicates. Different letters in the graph indicate significant differences (*p* < 0.05).

To explore the regulatory effects of OP on hepatic lipogenesis, mRNA expression levels of *SREBP-1c*, *ACC1*, *FASN*, and *DGAT2* were quantified. *SREBP-1c* serves as the master transcription factor that regulates the expression of genes involved in fatty acid and TG synthesis. As shown in Figure 3a, there was a 10-fold increase in *SREBP-1c* expression in the SO-Treated group, while treatment with OP50 significantly reduced expression by 36%. The levels of *SREBP-1c* were further decreased by 78 and 94% in the OP100 and OP250 groups, respectively, nearly restoring them to normal levels.

*ACC1* is essential for catalyzing fatty acid biosynthesis and serves as a significant regulator of lipogenesis (17). Figure 3b illustrated a 4.6-fold increase in *ACC1* expression in the SO-Treated group. Treatment with OP50 reversed this increase by 43%. *ACC1* levels were further diminished by 84 and 96% in the OP100 and OP250 groups, respectively, bringing them to approximately 25 and 80% lower than those in the control group. *FASN* is a key enzyme in the final step of fatty acid synthesis, converting free fatty acids into long-chain fatty acids and upregulating TG levels, which contributes to intracellular lipid accumulation (18). As shown in Figure 3c, sodium oleate significantly impacted *FASN* expression, with OP50 treatment resulting in a 17% reduction. *FASN* levels were further decreased by 79 and 94% in the OP100 and OP250 groups, respectively.

*DGAT2* plays a crucial role in fat digestion and absorption and is vital for converting diacylglycerol to TG (19). Figure 3d indicates a 4.8-fold increase in *DGAT2* expression in the SO-Treated group, which was significantly reduced by 34% following OP50 treatment. Similar decreases were observed in the OP100 and OP250 groups, with expressions approximately 79% lower than in the SO-Treated group and 69% lower than in the control group.

The *SREBP-1c/ACC1/FASN* pathway is a classical lipogenic cascade that tightly regulates cellular lipid synthesis. Under physiological conditions, a delicate balance is maintained between lipid production and clearance. However, when this balance is disrupted, excessive lipid accumulation occurs, promoting hepatic steatosis and metabolic dysfunction (20). Among the key regulators of this pathway, ACC1 is essential for maintaining the balance between lipogenesis and lipolysis in the liver (21). Under conditions of lipid oversynthesis, ACC1 is activated and dissociates from SREBP-1c, subsequently inducing the expression of downstream target genes such as FASN to promote fatty acid synthesis. The mRNA levels of *SREBP-1c*, *ACC1* and *FASN* —key regulators of fatty acid biosynthesis—are closely associated with lipid accumulation in hepatocytes (16). Furthermore, SREBP-1c acts as a transcription factor for both ACC1 and FASN, facilitating the conversion of excess carbohydrates into fatty acids via acetyl-CoA. Consequently, inhibition of SREBP-1c, ACC1, and FASN expression may alleviate hepatic steatosis and decelerate the progression of fatty liver disease. In addition, DGAT2, which is involved in triglyceride synthesis and lipid absorption, also contributes significantly to hepatic triglyceride accumulation (19). Collectively, OP treatment significantly reduced lipid accumulation. This reduction was accompanied by the coordinated downregulation of SREBP-1c and its downstream targets (ACC1, FASN, and DGAT2), consistent with the suppression of the lipogenic pathway.

### 3.4 OP treatment enhanced fatty acid β-oxidation genes in sodium oleate-treated HepG2 cells

**Figure 4:**
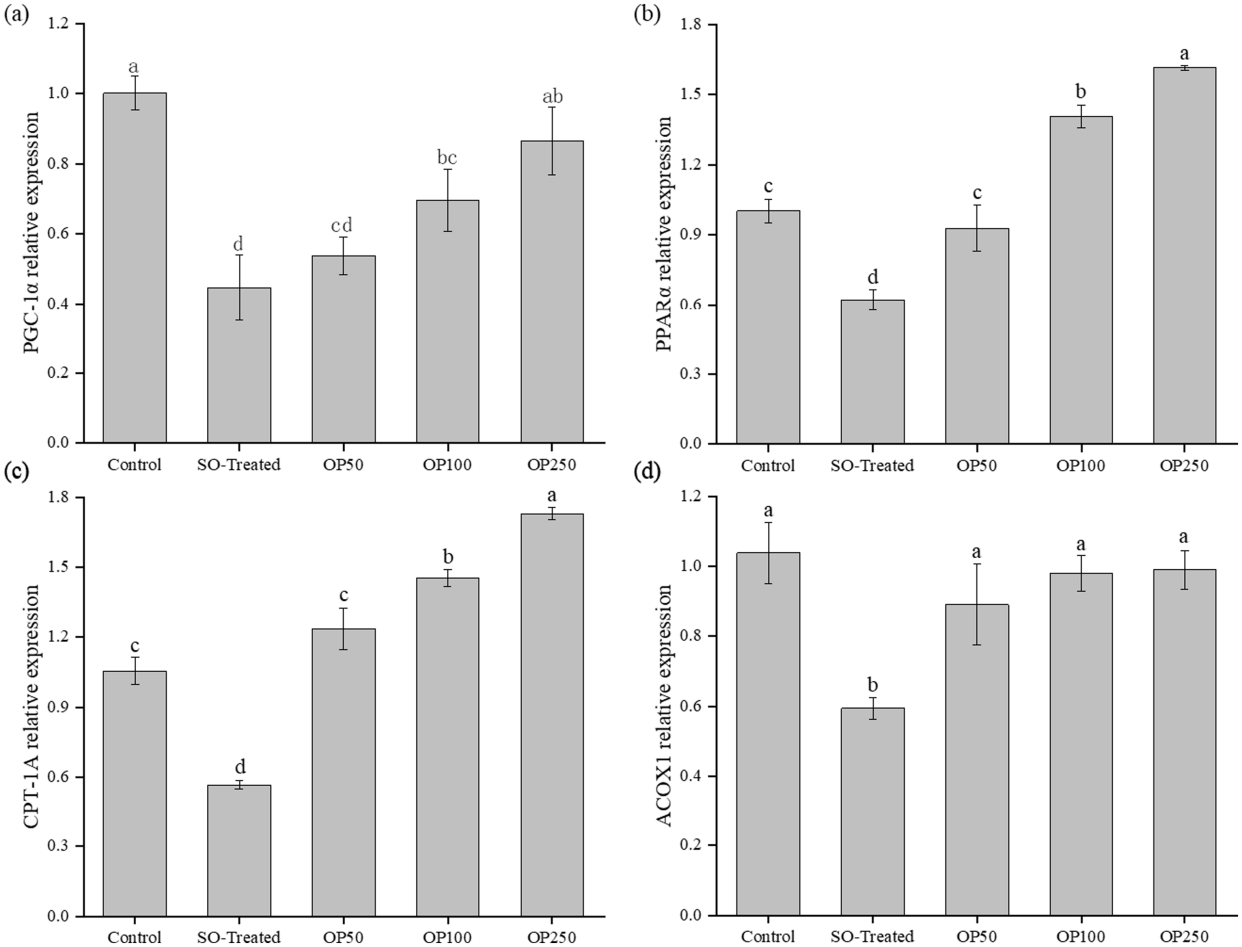
Effect of OP treatment on the relative expression of genes associated with fatty acid *β*-oxidation in SO-induced HepG2 cells. Relative transcript level of PGC-1α **(a)**, PPARα **(b)**, CPT-1A **(c)**, and ACOX1 **(d)**. Data in the bar graphs are expressed as mean ± SD and represent three biological replicates. Different letters in the graph indicate significant differences (*p* < 0.05).

To further elucidate the mechanisms underlying OP’s lipid-lowering effects, the mRNA expression of four major β-oxidation regulators—*PGC-1α*, *PPARα*, *CPT-1A*, and *ACOX1*—was analyzed following 24 h of treatment (Figure 4).

*PGC-1α* is a master transcription factor that regulates the expression of genes associated with β-oxidation (22). As shown in Figure 4a, *PGC-1α* expression was reduced by 56% in the SO-Treated group, indicating a significant change; however, treatment with OP50 did not reverse this decline. In contrast, *PGC-1α* levels increased by 57 and 95% in the OP100 and OP250 groups, respectively, compared to the SO-Treated group, although they remained significantly lower than in the control group.

The nuclear receptor PPARα plays a central role in mediating hepatic fatty acid metabolism. As illustrated in Figure 4b, *PPARα* expression decreased by 38% in the SO-Treated group, while OP50 treatment nearly restored it to normal levels. *PPARα* levels were further elevated in the OP100 and OP250 groups, which were approximately 41 and 61% higher than those in the control group, respectively. Figure 4c showed that *CPT-1A* expression decreased by 44% in the SO-Treated group; however, OP50 treatment reversed this decline, bringing levels back to normal. In the OP100 and OP250 groups, *CPT-1A* levels increased by approximately 45 and 73%, respectively, compared with the control group. In Figure 4d, sodium oleate significantly affected *ACOX1* mRNA expression, with OP50 treatment leading to a 51% increase. However, there were no significant changes in *ACOX1* levels in the OP100 and OP250 groups compared to the control group.

The homeostasis of lipid metabolism depends on a coordinated balance among lipogenesis, lipolysis, and fatty acid *β*-oxidation. The *PGC-1α/PPARα* signaling axis is a well-characterized pathway that enhances lipolysis and upregulates β-oxidation (23). *PPARα,* in particular, serves as a key transcriptional regulator of genes involved in fatty acid *β*-oxidation. Its activation has been shown to alleviate sodium oleate-induced lipid accumulation in HepG2 cells, leading to reduced triglyceride levels and overall lipid content. Under conditions of lipid overload, *PPARα* is activated and cooperates with *PGC-1α* to transactivate downstream targets such as *CPT-1A* and *ACOX1*, thereby promoting fatty acid β-oxidation (24). Thus, the expression of *PGC-1α, PPARα, CPT-1A,* and *ACOX1*—central regulators of *β*-oxidation—collectively contributes to the suppression of lipid accumulation in hepatocytes.

In the present study, treatment with 400 μM sodium oleate significantly decreased the expression of fatty acid β-oxidation genes, including *PGC-1α*, *PPARα*, *CPT-1A* and *ACOX1*. OP treatments effectively counteracted this suppression and, at concentrations of 100 and 250 μg/mL, elevated the expression of *PGC-1α*, *PPARα* and *CPT-1A* in a concentration-dependent manner compared with the SO-Treated group. Although *ACOX1* expression was also significantly increased by OP, no clear concentration-dependent effect was observed. These results indicate that OP treatment effectively restores the expression of β-oxidation-related genes. The concurrent upregulation of *PGC-1α*, *PPARα*, and their downstream targets (*CPT-1A* and *ACOX1*) suggests that OP may exert its effects by modulating this transcriptional network, although direct transcriptional activation remains to be verified.

### 3.5 OP increased AMPK phosphorylation in sodium oleate-treated HepG2 cells

To examine whether OP treatment is associated with changes in AMPK signaling, the protein expression levels of AMPK and p-AMPK were measured. AMPK serves as a key energy sensor and metabolic regulator that is rapidly activated during energy deprivation (25).

**Figure 5:**
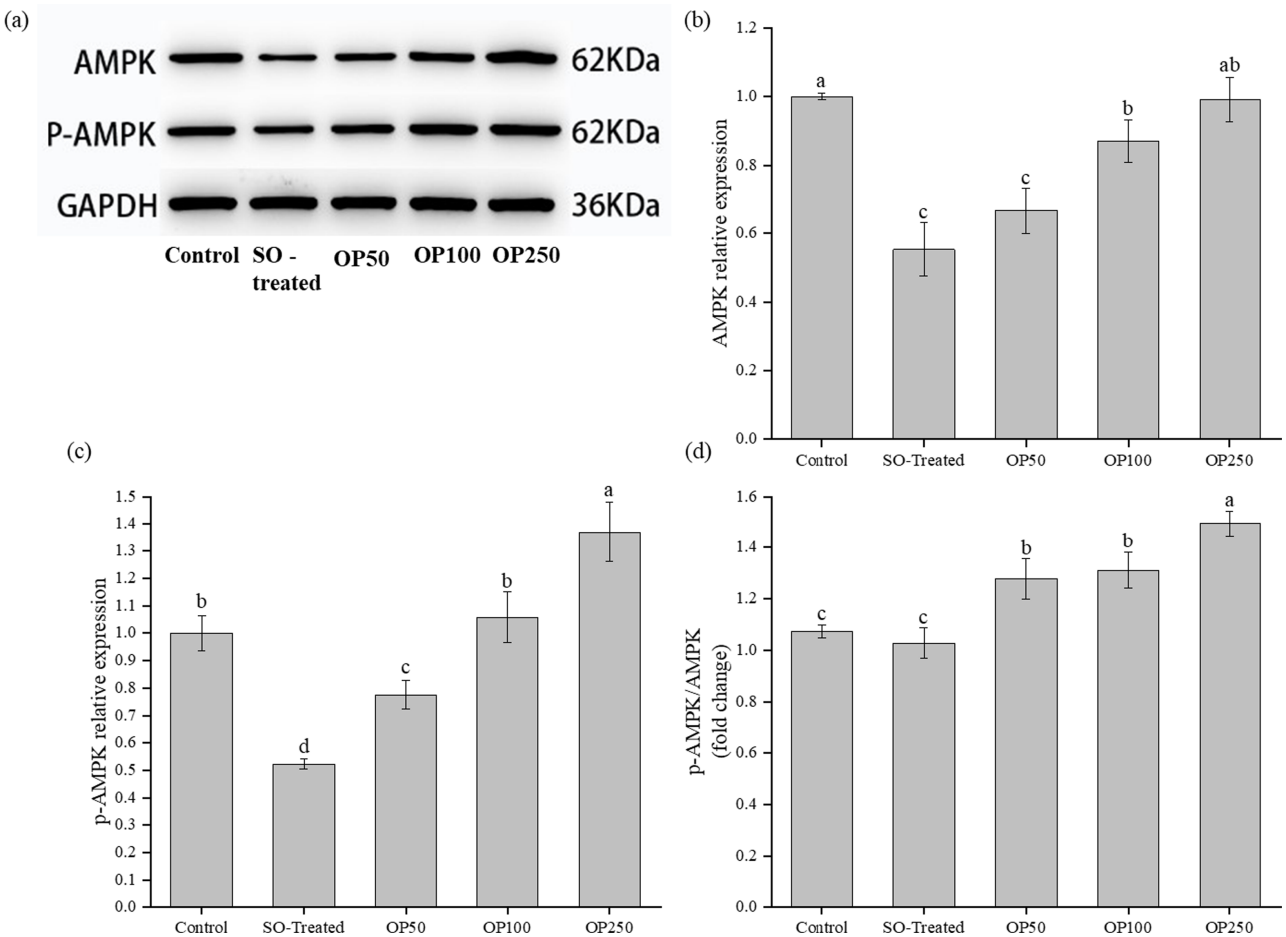
Effects of OP treatment on AMPK phosphorylation status in SO-induced HepG2 cells. **(a)** Representative immunoblot of total AMPK and p-AMPK; **(b)** Quantitative results of AMPK; **(c,d)** Quantitative results of p-AMPK **(c)** and the ratio of p-AMPK to AMPK. Data in the bar graphs are expressed as mean ± SD and represent of three biological replicates. Different letters in the graph indicate significant differences (*p* < 0.05).

As shown in Figure 5, Western blot analysis revealed a significant decrease in p-AMPK protein levels in the SO-Treated group compared to the Control group. In contrast, OP treatment considerably increased p-AMPK expression in a concentration-dependent manner. Consistent with these protein findings, mRNA levels of *PGC-1α* and *PPARα* were significantly downregulated in the SO-Treated group, while OP administration markedly restored their expression. Conversely, *SREBP-1c* mRNA expression was elevated in the SO-Treated group and significantly suppressed by OP treatment. We further assessed key genes involved in lipogenesis and fatty acid oxidation. The SO-Treated group exhibited upregulation of lipogenic genes (*ACC1*, *FASN* and *DGAT2*) and downregulation of fatty acid *β*-oxidation genes (*CPT-1A* and *ACOX1*; Figures 3b–d, 4c,d). OP treatment reversed these alterations, suppressing lipogenic gene expression and enhancing fatty acid oxidation-related transcripts. These changes occurred in parallel with increased AMPK phosphorylation.

These findings are consistent with previous reports that Chinese olive preparations reduce lipid accumulation and are accompanied by AMPK activation in hepatic models (9). AMPK, a heterotrimeric complex composed of a catalytic *α* subunit and two regulatory β and *γ* subunits, serves as a master regulator of cellular metabolism (26). Its activation alleviates lipid accumulation through dual mechanisms: suppression of lipogenesis and promotion of lipid catabolism. Phosphorylated AMPK inhibits lipogenesis by inactivating key regulators such as *SREBP-1c* and *ACC1* (27). *SREBP-1c* is a major transcription factor governing fatty acid desaturation and lipogenic enzyme expression, while *ACC1* catalyzes a critical step in *de novo* lipogenesis and concurrently suppresses fatty acid oxidation (28). In line with this, our study showed that OP enhanced p-AMPK expression while reducing mRNA levels of SREBP-1c, ACC1, and its target gene FASN in sodium oleate-induced HepG2 cells. These results align with reported AMPK-mediated repression of SREBP-1c/FASN signaling in NAFLD models.

Beyond suppressing lipogenesis, AMPK activation also promotes fatty acid *β*-oxidation via upregulation of *PPARα* and its target genes *CPT-1A* and *ACOX1*. Fatty acid oxidation plays an essential role in maintaining hepatic lipid homeostasis, and its dysregulation leads to excessive triglyceride accumulation and steatosis (27). *PPARα* is a nuclear receptor that transcriptionally activates fatty acid catabolic genes, thereby reducing hepatic lipid burden (29). *CPT-1A*, a *PPARα* target, acts as the rate-limiting enzyme in mitochondrial β-oxidation. In the present study, OP treatment significantly elevated the expression of *PPARα* and *CPT-1A* compared to the SO-Treated group.

Overall, OP reduced lipid accumulation in HepG2 cells and was accompanied by increased AMPK phosphorylation, decreased *SREBP-1c*-related lipogenic transcripts, and increased *PPARα*-related β-oxidation transcripts. Further mechanistic validation is required to establish pathway causality.

### 3.6 OP suppressed the overexpression of miR-122 and miR-21

**Figure 6:**
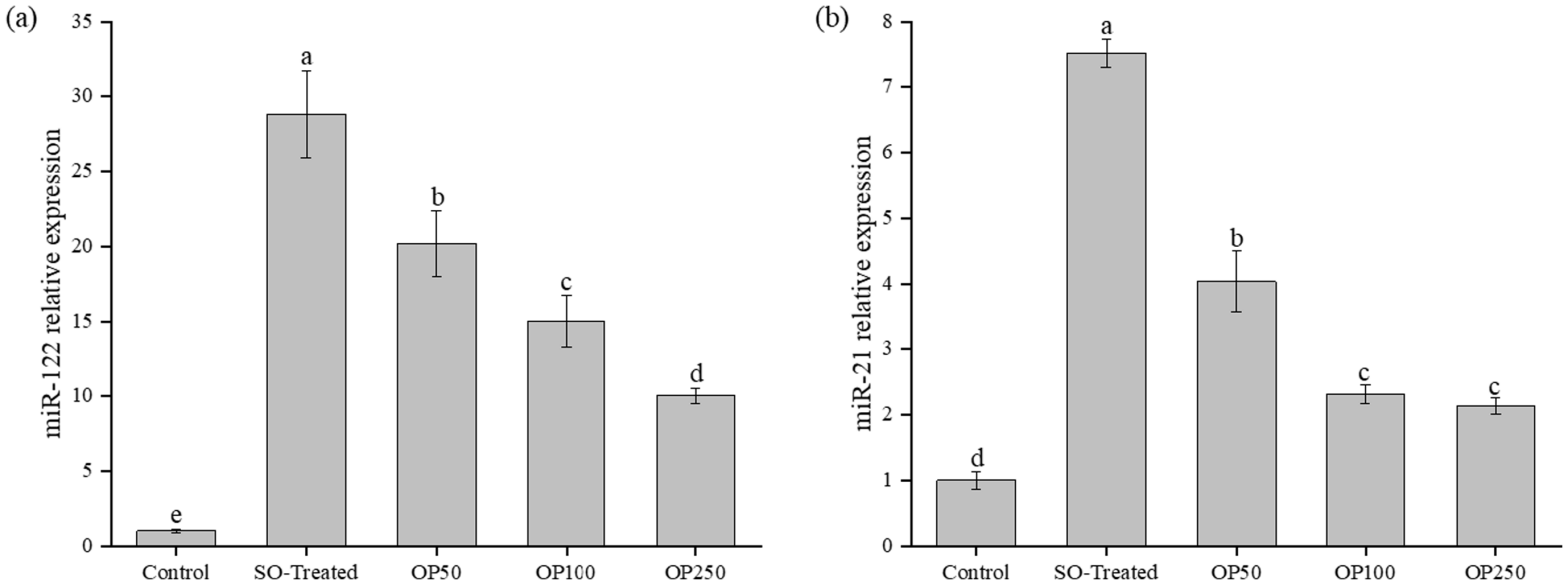
Effect of OP treatment on relative expression levels of miR-122 **(a)** and miR-21 **(b)** expression in SO-induced HepG2 cells. Data in the bar graphs are expressed as mean ± SD and represent of three biological replicates. Different letters in the graph indicate significant differences (*p* < 0.05).

MiRNAs are critical post-transcriptional regulators of lipid metabolism. To assess whether OP treatment is associated with miRNA changes, the expression levels of miR-122 and miR-21 were analyzed following 24 h of treatment (Figure 6).

In the present study, sodium oleate induction led to a 28-fold upregulation of miR-122 in the SO-Treated group (Figure 6a). This observation is consistent with recent findings by Hu et al. (30), who reported that high-fat diet and fatty acid exposure significantly upregulate hepatic miR-122 expression *in vivo* and *in vitro*. Furthermore, previous studies have shown that the overexpression of miR-122 facilitates lipid accumulation by targeting genes such as *SREBP-1c* and *DGAT2* (31). OP treatment significantly reversed this increase in a concentration-dependent manner, reducing miR-122 expression by 30, 49, and 67% in the OP50, OP100, and OP250 groups, respectively. Consistent with the reported role of miR-122, the mRNA levels of SREBP-1c and DGAT2 were also elevated upon sodium oleate induction (Figures 3a,d), accompanied by upregulation of downstream lipogenic genes. These results are consistent with prior reports linking miR-122 to lipogenic regulation (6). Because *SREBP-1c* and *DGAT2* have been reported as miR-122–related targets, the parallel changes observed here suggest a possible association between OP-induced miR-122 reduction and decreased lipogenic gene expression, however, causality remains to be experimentally validated.

We further examined the effect of OP on miR-21, another miRNA implicated in lipid homeostasis. As shown in Figure 6b, miR-21 expression increased by 7.5-fold in the SO-Treated group. OP50 treatment significantly reduced its level by 30%, and further decreases of 69 and 72% were observed in the OP100 and OP250 groups, respectively, though the response was not strictly concentration-dependent. *PPARα* has been identified as a downstream target of miR-21 (4). Its overexpression suppresses *PPARα* signaling and attenuates *β*-oxidation in hepatocytes. OP treatment decreased miR-21 levels and increased *PPARα* and related β-oxidation transcripts in parallel. These findings suggest a potential link between the modulation of miR-122/miR-21 and the lipid-lowering effects of OP. However, whether the downregulation of these miRNAs is the primary driver of the observed phenotypic changes requires further investigation using miRNA mimics or inhibitors.

### 3.7 Phenolic composition of OP and its association with lipid accumulation

Hepatic lipid accumulation is a hallmark of metabolic dysfunction and a key contributor to the development of fatty liver and related metabolic disorders. Previous studies have demonstrated that various natural extracts derived from fruits, vegetables, grains, and their by-products, such as pomegranate leaf extract (32), associated with improvements in lipid metabolism. These extracts are often characterized by complex phytochemical compositions, among which polyphenolic compounds represent a major constituent class.

In the present study, UPLC–MS/MS profiling identified 39 phenolic compounds in OP, comprising seven phenolic acids, six tannins, 18 flavonoids, and eight lignans and coumarins. The reported biological activities of representative compounds are summarized in Table 1. Given the compositional complexity of the extract, these constituents may collectively contribute to the lipid-associated effects observed in HepG2 cells; however, individual contributions cannot be distinguished based on the current experimental design.

**Table 1:** Major compounds detected in OP by UPLC-MS/MS analysis.

No.	CAS	Area (%)	Compound name	Chemical class	Molecular formula	Molecular weight	Biological activity
1	149–91-7	15.39	gallic acid	Phenolic acids	C_7_H_6_O_5_	170.02	Lipid lowering and antioxidant activities (32)
2	154,702–76-8	9.48	methyl brevifolincarboxylate	Phenolic acids	C_14_H_10_O_8_	306.04	Lipid lowering (12), anti-inflammatory (12) and anti-infection (34) activities
3	139,163–18-1	7.52	ellagic acid-4-o-xyloside	Tannins	C_19_H_14_O_12_	434.05	Inhibit cholinesterase (35) and improve lipid metabolism (33)
4	482–35-9	4.13	quercetin-3-o-glucoside	Flavonoids	C_21_H_20_O_12_	464.10	Antidiabetic and antioxidant (36), anticancer activity (37), antiproliferative activity (38), anti-inflammatory and hypolipidemic properties (39)
5	35,589–21-0	3.88	isohyperoside	Flavonoids	C_21_H_20_O_12_	464.10	Against H_2_O_2_-induced injury (40)
6	133–04-0	3.61	asarinin	Lignans and Coumarins	C_20_H_18_O_6_	354.11	Against 6-OHDA-induced cytotoxicity (41), inhibited MC cell proliferation (42) and inhibits immunological rejection (43)
7	99–24-1	3.27	methyl gallate	Phenolic acids	C_8_H_8_O_5_	184.04	Prevented hepatic damage (44), antioxidant and anti-HIV activities (45)
8	23,725–05-5	2.70	chebulic acid	Tannins	C_14_H_12_O_11_	356.04	Antioxidant activity (46), prevent endothelial cell dysfunction (47), prevents hepatic fibrosis (48), antineuroinflammation (49) and against cell oxidative stress (50), antiproliferative activity (51)
9	536–08-3	2.29	digallic acid	Phenolic acids	C_14_H_10_O_9_	322.03	Antioxidant activity (52)
10	831–61-8	1.84	ethyl gallate	Phenolic acids	C_9_H_10_O_5_	198.05	Inhibit lipid accumulation (53), antimicrobial (54) and anti-obese (55) activities
11	92–61-5	1.74	scopoletin	Lignans and Coumarins	C_10_H_8_O_4_	192.04	Antitumor activity (56), antioxidantion (57), inhibit hepatic steatosis (58) and anti-inflammation (59)
12	5,373-11-5	1.53	cynaroside	Flavonoids	C_21_H_20_O_11_	448.10	Antioxidantion (60), anticancer (61), inhibiting NLRP3 inflammasome activation (62), anti-inflammatory (63) and alleviate cell oxidative stress (64)
13	17,680–84-1	1.45	homoplantaginin	Flavonoids	C_22_H_22_O_11_	462.11	Anti-inflammatory (65) and hepatoprotective activities (66)
14	611–40-5	1.33	tectoridin	Flavonoids	C_22_H_22_O_11_	462.11	Against lung injury (67) and antioxidant activity (68)
15	3,934-84-7	1.30	3-o-methylgallic acid	Phenolic acids	C_8_H_8_O_5_	184.04	Antioxidant (69) and anticancer (70) activities
16	22,255–13-6	1.24	guaijaverin	Flavonoids	C_20_H_18_O_11_	434.08	Antiallergic rhinitis (71), inhibits pancreatic lipase (72) and lipid lowering activity (73)
17	482–36-0	1.17	hyperin	Flavonoids	C_21_H_20_O_12_	464.10	Antioxidation (74), anti-inflammation (75), improve the ovarian endocrine (76) and alleviate hyperglycemia (77)
18	572–30-5	1.14	avicularin	Flavonoids	C_20_H_18_O_11_	434.08	Anti-inflammation (78), alleviate insulin resistance (79) and inhibit lipid accumulation (80)
19	478–01-3	1.12	nobiletin	Flavonoids	C_21_H_22_O_8_	402.13	Antioxidant and anti-inflammatory (81) activities
20	23,094–69-1	0.91	corilagin	Tannins	C_27_H_22_O_18_	634.08	Anti-tumor (82), antihypertensive (83) and hepatoprotective (84) activities
21	60,976–49-0	0.90	geraniin	Tannins	C_41_H_28_O_27_	952.08	Alleviate osteoporosis (85)
22	529–51-1	0.81	azaleatin	Flavonoids	C_16_H_12_O_7_	316.06	Inhibit human glutaminyl cyclase (86)
23	578–74-5	0.73	apigenin-7-o-glucoside	Flavonoids	C_21_H_20_O_10_	432.11	Antibacterial, acetylcholinesterase-inhibitory (87), modulating gut microbiota (88), antibacterial (89), antioxidant and anti-inflammatory (90) activities
24	549–32-6	0.62	quercetin-3-o-xyloside	Flavonoids	C_20_H_18_O_11_	434.08	Immunomodulatory activity (91) and anti-pancreatitis activity (92)
25	481–53-8	0.55	tangeretin	Flavonoids	C_20_H_20_O_7_	372.12	Antibacterial (93), anti-inflammatory and antioxidant activities (94)
26	20,229–56-5	0.54	quercetin-4′-o-glucoside	Flavonoids	C_21_H_20_O_12_	464.10	Anti-depressant activity (95)
27	531–58-8	0.53	cichoriin	Lignans and Coumarins	C_15_H_16_O_9_	340.08	Antioxidative stress and lipid lowering activity (96)
28	531–75-9	0.52	esculin	Lignans and Coumarins	C_15_H_16_O_9_	340.08	Inhibit lipid peroxidation (97)
29	79,786–04-2	0.50	casuariin	Tannins	C_34_H_24_O_22_	784.08	Restored mitochondrial dysfunction (98)
30	40,246–10-4	0.45	glycitin	Flavonoids	C_22_H_22_O_10_	446.12	Inhibit lipid accumulation (99) and anti-lipid peroxidative activity (100)
31	120–08-1	0.40	scoparone	Lignans and Coumarins	C_11_H_10_O_4_	206.06	Alleviate mitochondrial dysfunction (101), lipid lowering (102) and antioxidant (103) activities
32	23,094–71-5	0.27	chebulagic acid	Tannins	C_41_H_30_O_27_	954.10	Not known
33	99–50-3	0.25	protocatechuic acid	Phenolic acids	C_7_H_6_O_4_	154.03	Lipid lowering (103) and antioxidant (104) activities
34	4,192-90-9	0.24	trilobatin	Flavonoids	C_21_H_24_O_10_	436.14	Alleviate abnormal lipid metabolism and antioxidantion (105)
35	2,798-20-1	0.22	gardenin b	Flavonoids	C_19_H_18_O_7_	358.11	Not known
36	17,803–12-2	0.21	justicidin c	Lignans and Coumarins	C_22_H_18_O_7_	394.11	Not known
37	25,001–57-4	0.21	justicidin a	Lignans and Coumarins	C_22_H_18_O_7_	394.11	Not known
38	5,041-82-7	0.20	isorhamnetin-3-o-glucoside	Flavonoids	C_22_H_22_O_12_	478.11	Inhibit adipogenesis (106)
39	10,597–60-1	0.20	hydroxytyrosol	Phenolic acids	C8H10O3	154.06	Antioxidantion (107) and alleviates lipid accumulation (108)
40	63,358–12-3	0.19	isolariciresinol-9′-o-glucoside	Lignans and Coumarins	C_26_H_34_O_11_	522.21	Not known

Among the detected compounds, 17 constituents, including gallic acid, methyl brevifolincarboxylate, and ellagic acid-4-O-xyloside, showed relatively high signal responses in the UPLC–MS/MS analysis and were therefore discussed as representative features of the phenolic profile. Previous studies have reported that some of these compounds are associated with lipid metabolism–related cellular processes, such as AMPK phosphorylation and regulation of lipogenic gene expression (12, 33). In the present study, such findings provide background information rather than direct mechanistic evidence linking individual compounds to the observed lipid-associated effects.

Consistent with the observations of Yeh et al. (9), our results demonstrate that treatment with a Chinese olive extract is associated with reduced lipid accumulation in hepatocytes. Compared with previous studies, the present work provides additional phytochemical characterization of the extract used, allowing discussion of candidate phenolic features that may be relevant to the observed phenotype, while acknowledging that this study does not establish which compounds are necessary or sufficient to elicit the effect.

It should also be recognized that the phytochemical composition of Chinese olive can vary with cultivar, geographic origin, and cultivation conditions. Here, we focused on the commercially dominant cultivar ‘Tanxiang No.1’ to ensure experimental consistency, and the reported phenolic profile should therefore be interpreted as representative of this specific cultivar. Broader sampling across multiple origins, together with quantitative analyses, will be valuable in future studies to further characterize compositional variability and support standardization of polyphenol-rich Chinese olive extracts.

## 4 Conclusion

**Figure 7:**
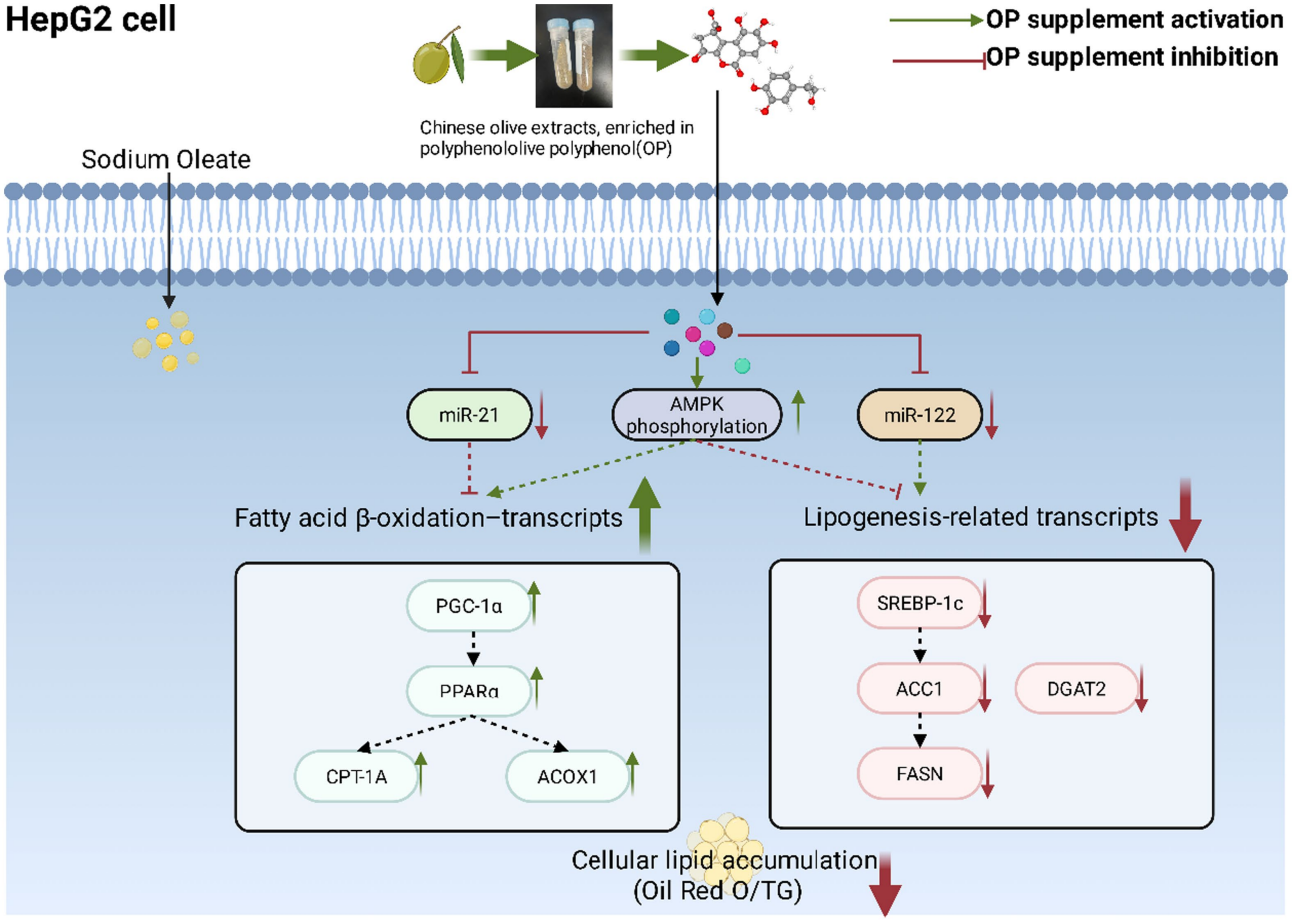
Schematic summary of observed associations between OP treatment, AMPK phosphorylation, miRNA expression, and lipid metabolism–related transcripts in SO–treated HepG2 cells. Solid arrows indicate observed changes in this study. Dashed arrows indicate putative associations inferred from previously report. Arrows (↑/↓) indicate the direction of change relative to SO-treated group.

In conclusion, treatment with polyphenol-rich Chinese olive extracts were associated with attenuation of lipid accumulation in sodium oleate-treated HepG2 cells, accompanied by transcriptional changes related to both lipogenesis and fatty acid *β*-oxidation. Extract treatment was also associated with increased AMPK phosphorylation and concurrent alterations in miR-122 and miR-21 expression, together with changes in lipid metabolism–related genes (Figure 7). The extract exhibited a complex phenolic composition, including methyl brevifolincarboxylate and ellagic acid derivatives, however, the specific bioactive constituents and the causal relationships among the observed molecular events remain to be elucidated. Further mechanistic studies are required to clarify the contributions of individual compounds and signaling pathways. Overall, these findings provide descriptive evidence linking Chinese olive extract treatment to coordinated lipid-associated molecular changes in hepatocytes and offer a basis for future mechanistic and translational investigations into its potential application in functional food development.

## Data Availability

The raw data supporting the conclusions of this article will be made available by the authors, without undue reservation.
