# Supplementary material for: Polyphenol-rich Chinese olive extracts attenuate lipid accumulation in HepG2 cells, accompanied by AMPK phosphorylation and miRNA alterations
Source: Front Nutr. 2026 Feb 11;13:1749622. doi: 10.3389/fnut.2026.1749622 (PMC12932180; doi:10.3389/fnut.2026.1749622)
Supplement: Supplementary file 1 [file Table_1.DOCX]

**Supplemental Materials**

**Table S1. Sequences of the primers used in RT-qPCR.**

| Gene name | NCBI ID | Primer Sequence (5′-3′) | |
| --- | --- | --- | --- |
| *GAPDH* | 2597 | Forward | CAAATTCCATGGCACCGTCA |
|  |  | Reward r | GGACTCCACGACGTACTCAG |
| *SREBP-1c* | 6720 | Forward | CGGAACCATCTTGGCAACAGT |
|  |  | Reward | CGCTTCTCAATGGCGTTGT |
| *FASN* | 2194 | Forward | AACCTGCACTTCCATAGCCC |
|  |  | Reward | GATGATGTGCACGTTGGAGC |
| *ACC1* | 31 | Forward | TCACACCTGAAGACCTTAAAGCC |
|  |  | Reward | AGCCCACACTGCTTGTACTG |
| *DGAT2* | 84649 | Forward | ATTGCTGGCTCATCGCTGT |
|  |  | Reward | GGGAAAGTAGTCTCGAAAGTAGC |
| *PPARα* | 5465 | Forward | tatggctgagaagacgctgg |
|  |  | Reward | gaattccgtgagctccgtga |
| *PGC-1α* | 10891 | Forward | TGAAGACGGATTGCCCTCATT |
|  |  | Reward | GCTGGTGCCAGTAAGAGCTT |
| *CPT-1* | 1374 | Forward | catacgaggcctccatgacc |
|  |  | Reward | tgcttctcagacgccaactt |
| *ACOX1* | 51 | Forward | gggcgcatacatgaaggaga |
|  |  | Reward | agccatccgacatgcttcaa |

**Table S2. Sequences of miRNA primers used in RT-qPCR.**

| Gene names | Forward primer | Reverse primer |
| --- | --- | --- |
| U6 | CAAATTCGTGAAGCGTTCCA | AGTGCAGGGTCCGAGGTATT |
| U6 Reverse Transcription Primers | GTCGTATCCAGTGCAGGGTCCGAGGTATTCGCACTGGATACGACAAAATA | |
| miR-122 | AACACCAGTCGATGGGCTGT | AGTGCAGGGTCCGAGGTATT |
| miR-122 Reverse Transcription Primers | GTCGTATCCAGTGCAGGGTCCGAGGTATTCGCACTGGATACGACTGTCAG | |
| miR-21 | CGCCATTATCACACTAAATAGCTACTG | AGTGCAGGGTCCGAGGTATT |
| miR-21 Reverse Transcription Primers | GTCGTATCCAGTGCAGGGTCCGAGGTATTCGCACTGGATACGACGCCTAG | |
