# Supplementary material for: Polyphenol-rich Chinese olive extracts attenuate lipid accumulation in HepG2 cells, accompanied by AMPK phosphorylation and miRNA alterations
Source: Front Nutr. 2026 Feb 11;13:1749622. doi: 10.3389/fnut.2026.1749622 (PMC12932180; doi:10.3389/fnut.2026.1749622)
Supplement: Supplementary file 2 [file Table_2.DOCX]

**Response to reviewers**

Reviewer #1: The manuscript presents an intriguing study investigating the lipid-lowering effects of Chinese olive (CO) polyphenols (OP) in a sodium oleate (SO)-induced lipid accumulation model using HepG2 cells.

Here are several comments and suggestions that could further strengthen the manuscript, as outlined below:

1. It is rather difficult to standardize the natural products, so I believe it would be better to collect different sources of Chinese olives, at least from 3 different sources (n=3), and analyze the components.

***Response:***

Thank you very much for your review.

We agree that analyzing olives from multiple geographical sources would provide a more comprehensive phytochemical profile. The primary aim of this study was to characterize the lipid-associated cellular responses and compositional features of polyphenol-rich extracts from the specific cultivar 'Tanxiang No.1', which is the commercially dominant cultivar in the main production region. To ensure experimental consistency and reproducibility, we used a single, well-characterized batch for all extractions and cell treatments. We have also added a statement to the end of Section 3.7 acknowledging this limitation and proposing that multi-origin compositional comparisons and biological validations will be key directions for future studies.

(2) The determination of non-toxic concentrations for OP and SO shown in Fig. 1 is appropriate and clearly presented. However, it is unclear whether the concentrations of OP used in the experiments reflect physiologically relevant levels. Could the authors please verify and discuss this aspect?

***Response:***

Thank you very much for your comments.

The concentrations used (50–250 μg/mL) were selected based on the initial cytotoxicity screenings to maximize the observable molecular signal *in vitro* without inducing cell death. We acknowledge that these *in vitro* concentrations may be higher than the physiological relevant levels achieved through diet. Therefore, we have revised the Section 3.1 to clearly state that our findings reflect cellular responses under experimental conditions and should not be directly interpreted as equivalent to *in vivo* exposure. Future studies will be required to determine bioavailability and effective physiological doses *in vivo*.

(3) In Fig. 2a, the OP250 treatment appears to have fewer cells than the other groups. Could the authors clarify whether the co-treatment of OP with SO has a synergistic or additive effect on cell viability? To avoid possible false positives due to reduced cell confluence at higher OP concentrations with SO, it would be helpful to include cell viability assays under combined OP and SO treatments.

***Response:***

We appreciate the reviewer's careful observations.

We would like to clarify that the apparent reduction in cell density in the OP250 image was due to the selection of a specific field of view to highlight lipid droplet contrast. To address potential confounding by cell number, we have performed a cell-viability assay under the co-treatment of OP with SO (Fig. 1c). The results show no significant reduction in viability across the OP concentrations used, indicating that the lipid-lowering effects are not attributable to decreased cell number. To avoid confusion, we have replaced Fig. 2a with representative images captured from randomly selected fields at the same magnification and exposure settings.

(4) The manuscript discusses several key pathways involving miR-122, SREBP-1c, DGAT2, PPARα, and AMPK. While the expression data are convincing, additional mechanistic experiments would strengthen the causal links. For instance, in Fig. 4, I recommend including reporter gene assays or other comparable experiments to validate the transcriptional activities of PPARα and PGC-1α on downstream targets such as CPT-1A and ACOX1. Similarly, in Fig. 3, if SREBP-1c is proposed as a transcription factor regulating ACC1 and FASN, reporter assays or equivalent experiments should be considered to confirm this regulatory relationship.

***Response:***

Thank you for the helpful suggestion.

We agree that reporter assays or comparable transcriptional activity assays would strengthen causal inference regarding transcriptional regulation. Due to scope and resource constraints, however, we did not perform additional reporter experiments in the current revision. Therefore, we revised the manuscript to avoid causal statements and describe our findings as consistent with the established SREBP-1c- and PPARα-related regulatory networks. We have also added this point explicitly as a limitation and a priority for future mechanistic validation.

(5) To demonstrate that miR-122 is a target of OP and mediates its effects on SREBP-1c and DGAT2, experiments involving the overexpression of miR-122 (e.g., transfection of miR-122 expressing vector) would be valuable to observe whether related gene expressions are modulated by miR-122. AMPK and PPARα expression following miR-122 modulation also would provide further mechanistic clarity.

***Response:***

Thank you for this insightful comment.

We agree that miR-122 overexpression experiments would be required to establish necessity/causality. In the current study, we report that OP treatment is accompanied by reduced miR-122 levels together with coordinated changes in lipogenesis-related transcripts, which is consistent with published literature. We have revised the text to avoid causal claims and have explicitly identified miRNA modulation experiments as a key limitation and future direction.

(6) Please include a schematic figure summarizing the proposed mechanism by which OP regulates lipid metabolism, which would greatly enhance the manuscript’s impact.

***Response:***

Thank you for your suggestion! The schematic summary of observed associations as Fig.7 has been supplemented.

(7) Other suggestions

- In Figure 2a, please add a scale bar.

***Response:***

We deeply appreciate these critical suggestions.

A scale bar has now been added to Fig. 2a (400×) to improve clarity.

(8) Please justify the choice of Duncan’s multiple range test over other tests such as Tukey’s.

***Response:***

Thank you for the suggestion.

We acknowledge that Duncan's multiple range tests are less conservative than Tukey's HSD. To strengthen the statistical rigor, we have re-analyzed the multiple comparisons using Tukey's. The overall significance patterns and conclusions remain unchanged.

(9) In Figure 2b, OP250 appears to share the same statistical group (letter “c”) with the control but is labeled as “d.” Please verify this labeling for accuracy.

***Response:***

Thank you for your suggestion.

We have re-verified the statistical labels in Fig. 2b using IBM SPSS Statistics version 26. The difference between the OP250 group and the Control group is statistically significant; therefore, the labeling of "d" and "e" is correct. We have clarified the error bars and notation in the figure legend to ensure clarity.

(10) To improve clarity in graphs, consider adding a row below indicating the presence or absence of sodium oleate treatment, rather than using the term “model.”

***Response:***

Thank you for this valuable suggestion! We have replaced "Model" with "SO-treated" in the figures for clarity.

(11) Please provide detailed information about the antibodies used in the methods section, particularly those for AMPK and its phosphorylated forms.

***Response:***

We deeply appreciate these critical suggestions.

Detailed antibody information was added in the Materials and Methods section with the specific details for all antibodies used.

Reviewer #2: Comments to the Author

The manuscript of W. Bai provides evidence that oleic acid-enhanced changes in HepG2 cells are suppressed by Chinese olive polyphenols (OP) in a concentration-dependent manner. The findings are of potential interest to the audience of the journal, are novel, and supported by the data. Minor recommendations for revision are listed below.

(1) The authors use the term "dose-dependent" to describe the effects of OP on their model system, which is cultured HepG2 cells. It would be more accurate to use the term "concentration-dependent", since using "dose" implies an in vivo study

***Response:***

Thank you for this valuable suggestion.

We have replaced "dose-dependent" with "concentration-dependent" throughout the manuscript.

(2) In methods section 2.6, the authors state, "Intracellular TG content was assessed using a commercial assay kit." but no further information is provided. Please include the source of the assay kit and describe the method in sufficient detail so it can be replicated by others.

***Response:***

We have added the manufacturer (Nanjing Jiancheng Bioengineering Institute, Nanjing, China) and a detailed description of the TG assay procedure in Section 2.6.

(3) Please describe the source of the antibodies used in this study. Also include sufficient detail to permit replication (e.g., the phosphorylation site on AMPK is assumed to be P-Thr172, but it should be stated explicitly in the manuscript).

***Response:***

Thank you very much for your review and suggestion.

Detailed antibody information was added in the Materials and Methods section with the specific details for all antibodies used. We have also explicitly stated that the p-AMPK antibody targets the Thr172 phosphorylation site.

(4) In methods section 2.8, the authors state that primers used for real time PCR analysis are included in table S1. I may have missed downloading this file, but please confirm with the journal that the supplemental table with the primers gets included in a final version, if accepted.

***Response:***

Table S1 and S2, which contain primer sequence, were included as a separate Supplementary Material file, and their inclusion in the submission system was confirmed.

(5) In methods section 2.10, the authors state that they used Duncan's multiple range tests (DMRT) as a method for statistical analysis. DMRT analysis is a relatively weak method for determining significance compared to Tukey. Please confirm that DMRT is acceptable for this journal.

***Response:***

Thank you very much for your suggestion.

We acknowledge that Duncan's multiple range tests are less conservative than Tukey's HSD. To strengthen the statistical rigor, we have re-analyzed the multiple comparisons using Tukey's. The overall significance patterns and conclusions remain unchanged.

(6) Recommend acknowledging the prior work of Hu et al., Front. Physiol. Vol 13 (2022), https://doi.org/10.3389/fphys.2022.803445.

***Response:***

We have cited the recommended work (Hu et al., *Front. Physiol.* 2022) in the Section 3.6. This study provided important context by demonstrating that fatty acid exposure significantly upregulated hepatic miR-122 levels and that reducing miR-122 conferred hepatoprotective effects against lipid-induced damage, which aligns with our observations in the sodium oleate-induced model.

Reviewer #3: In the manuscript titled, “Chinese Olive Polyphenols Alleviate Hepatic Lipid Accumulation via AMPK Activation and microRNA-Mediated Pathways in HepG2 Cells,” the authors tested the effects of a polyphenol-containing extract of Chinese olive on fatty acid (oleic acid)-induced lipid accumulation in a hepatocellular carcinoma cell line. In their study, the authors used an in vitro model of lipid accumulation by treating HepG2 cells with oleic acid. Varying concentrations of the Chinese olive extract were then applied to this model to assess its effect on intracellular lipid content and AMPK activity, as well as relative transcript levels of genes involved in lipid metabolism. The authors also characterized the (poly)phenolic constituents of the extract used for their experiments using UPLC–MS/MS. Although the assays used to generate the data were performed adequately, this study is laden with major flaws or shortfalls and errors in data interpretation. Please see comments below.

Major comments:

“Correlation does not imply causation.” The authors repeatedly failed to take this into consideration when interpreting the results, and thus, conclusions made by the authors (e.g., “Chinese olive polyphenols alleviate hepatic lipid via AMPK activation and microRNA-mediated pathways in HepG2 cells accumulation”) are not invalid.

a. For example, OP treatment may result in the concentration-dependent decreases in miRNA-122 and 21 levels in HepG2 cells. This establishes correlation. However, this does not imply that the changes observed with these miRNAs are responsible for (i.e., causing) the reduction in intracellular lipid content. Same thing goes for AMPK activity.

***Response:***

Thank you very much for this critical comment.

We fully agree that the observed reductions in miRNAs expression, as well as the increase in AMPK phosphorylation following Chinese olive extract treatment, represent associative observations rather than evidence of causality.

In the revised manuscript, we have carefully revised the Abstract, Discussion, and Conclusion to clearly state that both miRNA modulation and AMPK phosphorylation were accompanied by or concurrent with, but not demonstrated to be responsible for, the observed reduction in intracellular lipid accumulation. All causal or mechanistic phrasing has been replaced with descriptive and correlative language.

b. To establish causal relationships between miRNAs or AMPK and OP-induced reduction in lipid accumulation, for instance, one must 1) alter miRNA levels (e.g., overexpression of miRNA mimics) in the presence of OP and 2) inhibit AMPK activity (e.g., via a selective inhibitor) in the presence of OP and see whether the effect of OP is abolished.

***Response:***

Thank you very much for your review and suggestion.

We agree that targeted intervention experiments, such as AMPK inhibition or miRNA gain- or loss-of-function analyses, are necessary to establish causality. However, these experiments were beyond the scope of the present study. Accordingly, we have revised the manuscript to clearly state this limitation and have avoided making mechanistic claims.

c. Unless the mechanistic experiments suggested above are included, the authors must not make any solid and bold conclusions, such as the one stated by the title, for the authors only observed the correlation (not causation) between the observed changes and OP treatment. This represents a major flaw with the current study.

***Response:***

We fully agree with the reviewer’s concern. To address this issue, we have revised the title and all relevant sections of the manuscript to adopt a descriptive and associative tone. Specifically, causal terms such as “via” and “mediated by” have been removed, and the revised title now emphasizes observed associations rather than mechanistic conclusions.

Previously, Yeh et al. (PMID: 29348600) published a similar yet more extensive (using both in vitro and in vivo models) report. The findings of the current study support this previously published report. However, in contrast to the authors’ claims, the current study remains descriptive and does not provide any mechanical insight regarding the effects of Chinese olive extract on hepatic lipid content (see the comment above).

***Response:***

Thank you very much for your review.

We agree that our findings are consistent with and supportive of previous reports, including Yeh et al. (PMID: 29348600). We have revised the Introduction to explicitly position our study as an extension of their work. Our study adds value by providing a detailed UPLC-MS/MS characterization of the specific phenolic profile of the 'Tanxiang No.1' cultivar and by identifying the specific miRNA alterations (miR-122 and miR-21) that accompany the lipid-lowering effects, which were not explored in the previous report.

The authors did not provide any evidence to support their claim that the polyphenolic content of Chinese olive is mediating the protective effect of the extract against intracellular lipid accumulation.

a. Based on the UPLC-MS/MS analysis, the (poly)phenolic components, in combination, seem to represent a major detected constituent (~70%). However, without proper standards, their analysis remains semi-quantitative or qualitative at best, and the concentration or molar fraction of each constituent is unknown. Thus, it is unclear how much (in molar mass) the (poly)phenolic components are represented in the extract used for this study. For this reason, it is misleading to make statements, such as “Chinese olive polyphenols alleviate hepatic lipid accumulation…” To make such definitive statements, one would need to 1) purify and isolate Chinese olive polyphenols and test their efficacy directly or 2) get rid of polyphenols from the extract and show that the effects of the extract (against lipid accumulation) are abolished.

***Response:***

Thank you very much for this important and detailed comment.

We agree that the UPLC–MS/MS analysis performed in the present study provides qualitative and semi-quantitative information on the phenolic composition of the extract, rather than absolute quantification of individual compounds. We have therefore removed all definitive statements implying that polyphenols alone are responsible for the attenuation of lipid accumulation. Instead, we now consistently refer to polyphenol-rich Chinese olive extracts or Chinese olive extract, enriched in polyphenols (OP) and describe the phenolic profile as a major compositional feature, without assigning exclusive causality. We further clarify that purification or depletion experiments would be required to directly establish the contribution of polyphenols.

b. In other words, it is clear that the extract used in the study contains a myriad of polyphenols, but it should not be concluded that the polyphenolic content (only) mediates the effects observed in the study. Unless the authors make major revisions to this statement, their conclusions are invalid.

***Response:***

We fully agree with the reviewer that the extract used in this study represents a complex mixture of bioactive constituents and that it cannot be concluded that polyphenolic compounds alone mediate the observed lipid-associated effects. In response to this comment, we have revised the Section 3.7 and Conclusion to explicitly acknowledge this limitation and to avoid exclusive attribution of bioactivity to polyphenols.

c. The extraction method authors described does not match any of the methods mentioned in the previous study they referenced (Nayak et al. PMID: 25977057). Authors would need to 1) provide a rationale for using a particular extraction method or 2) provide experimental evidence showing that the method used in the current study is optimal for extracting (poly)phenolic content of the fruit.

***Response:***

Thank you for this important comment. We have clarified that the extraction protocol used in this study is an adaptation of the method described by Nayak et al. (PMID: 25977057), not a direct replication. We have revised Section 2.2 to explicitly provide the rationale for our specific methodology.
